# Transcriptional profile of *Glaesserella parasuis* in swine serosal and joint fluids

**DOI:** 10.3389/fvets.2025.1452973

**Published:** 2025-04-25

**Authors:** Daniel W. Nielsen, Kaitlyn M. Sarlo Davila, Susan L. Brockmeier, Samantha J. Hau

**Affiliations:** ^1^Ruminant Diseases and Immunology Research Unit, National Animal Disease Center (USDA-ARS), Ames, IA, United States; ^2^Virus and Prion Research Unit, National Animal Disease Center (USDA-ARS), Ames, IA, United States

**Keywords:** transcriptional, RNA-Seq, swine, *Glaesserella parasuis*, serosal, joint, Glässer's disease

## Abstract

*Glaesserella parasuis* is the causative agent of Glässer's disease and contributes to significant post-weaning mortality in the swine industry. Glässer's disease is characterized by meningitis, polyserositis, and polyarthritis. Previous work has examined transcriptomic differences of *G. parasuis* when inoculated into different *in vitro* conditions, lung explants, or the lung *in vivo* following intratracheal challenge. However, it is still unknown how the transcriptome of *G. parasuis* may change to cause polyserositis or polyarthritis. Here, we incubated *G. parasuis* in acellular joint or serosal fluid for 3 and 12 hours to better understand transcriptional changes in the joint or serosal compartment. When *G. parasuis* serovar 5 strain 29755 was incubated in host fluid for 3h, cell wall, membrane, and envelope biogenesis genes were downregulated compared to *G. parasuis* incubated in PBS. In contrast, translation, ribosomal structure, and biogenesis and carbohydrate transport and metabolism were upregulated in the host fluid compared to PBS. Additionally, there were eleven differentially expressed genes with an unknown function shared between the acellular joint and serosal fluid at the 3h timepoint compared to PBS. When comparing the differences between the host fluids from 12 to 3h and the host fluids at 3h compared to PBS, this study found sixteen genes with inverse expression patterns. An investigation into the hypothetical genes identified and the nineteen shared genes in all comparisons may provide further knowledge about the pathogenesis of *G. parasuis*, which may be useful in developing interventions against Glässer's disease.

## Introduction

*Glaesserella parasuis* (*G. parasuis*), formerly *Haemophilus parasuis*, is both a commensal of the swine nasal cavity and the causative agent of Glässer's disease. In the United States, Glässer's disease is considered one of the main infectious problems in the nursery phase (1). To cause disease, *G. parasuis* enters through the nasal cavity, and subsequently, the bacteria may be isolated from the lungs (1). For virulent strains, such as serovar 5 strain 29755, the bacteria traverses the epithelial barrier and enters the blood stream, and it can be isolated from internal organs (1). Experimentally, strain 29755 is highly virulent and resulted in mortality of 87.5% of Caesarian-derived, colostrum-deprived (CDCD) piglets following intranasal challenge (2). At necropsy, the strain could be isolated from the pleura, pericardium, peritoneum, joint, meninges, and via lung lavage of infected animals (2).

Previous work by Hau et al. (3) examined transcriptomic differences for strain 29755 when grown in broth or on agar. The work found the transcription of most previously described virulence-associated genes were comparable between broth and agar, except for *ompA, vapD*, and several protease genes (3). Other work by Alvarez-Estrada et al. (4) investigated transcriptomic differences when *G. parasuis* serovar 5 strain Nagasaki was subjected to *in vitro* conditions of high temperature and iron restriction, which mimic conditions during host infection. Bello-Ortí et al. (5) identified upregulation of putative or known pathogenesis or virulence genes when the transcriptome of *G. parasuis* strain Nagasaki was compared from both lung explants and the lungs to growth on a conventional agar plate. Their findings identified an upregulation of genes involved in carbon acquisition, iron binding and pathogenesis, ABC transporters, virulence-associated autotransporters, and several hypothetical proteins (5).

However, work examining transcriptional differences of a *G. parasuis* in a mock *in vivo* scenario related to the joint or serosal fluid has not yet been completed. As strain 29755 may be isolated from the pleura, pericardium, peritoneum, and joints of CDCD pigs (2), we assessed transcriptomic differences when this strain was grown in acellular serosal and joint fluid. Additionally, we examined transcriptional differences associated with establishing infection by comparing both the acellular joint and serosal fluids at 3 and 12h post-exposure. We hypothesize that differentially expressed genes in the host fluids may play an active role in the development of arthritis and polyserositis.

## Materials and methods

### Sample collection

Thirteen healthy, cesarean derived, colostrum deprived pigs were euthanized for sample collection. Serosal fluid was collected from the pericardial, pleural, and peritoneal cavities by syringe. Joint fluid was obtained from hocks and elbows. Skin was removed from over the joint and fluid collected by syringe and needle. Fluid was pooled from the thirteen animals and syringe filtered with a 0.22 μm filter. Samples were frozen at −80°C until use.

Inocula were prepared by growing *G. parasuis* strain 29755 (serovar 5) on BHI+ NAD agar and suspending the bacteria in PBS. The inocula were pelleted and ~1.56 x 10^9^ CFU was combined with 500 μL of either serosal fluid, joint fluid, or PBS in a 1.5 mL tube. The cultures were incubated for 3 or 12h at 39°C to represent mid-to-late logarithmic growth of *G. parasuis*. Cultures were immediately pelleted and resuspended in 600 μL of TRI Reagent (ThermoFisher Scientific, Waltham, MA). The tubes were vortexed for 60 s and incubated at room temperature for 4 min before being frozen at −80°C.

### RNA extraction

To extract total RNA from samples, chloroform was added in a 1:5 ratio to TRI Reagent-cell mixture. The mixture was centrifuged at 4°C. RNA was extracted with the miRNAeasy miRNA Kit (Qiagen, Germantown, MD) following the manufacturer's instructions. Following RNA extraction, the 50 μL containing the extracted RNA was treated with TURBO DNA-free kit (ThermoFisher Scientific). RNA quality was assessed using the Agilent 2100 Bioanalyzer RNA 6000 Nano kit (Agilent Technologies, Santa Clara, CA).

### RNA-Seq library preparation and sequencing

Library preparation was performed at the Iowa State University Sequencing Facility (Ames, IA) on the 36 submitted samples, which represented six biological replicates per tested condition. Briefly, stranded total RNA-Seq libraries were prepared by depleting bacterial rRNA using the NEBNext Ultra II Directional RNA kit (New England BioLabs Inc., Ipswich, MA). Each sample was run on two different lanes on an Illumina NovaSeq 6000 instrument (San Diego, CA). The generated reads were 100 bp single end reads.

### Data analysis

Reads were trimmed using Trimmomatic 0.38, and the post-trimming read quality was assessed with FastQC v. 0.11.5 (6, 7). The Bowtie2 aligner (v. 2.4.5) was used to map trimmed reads to the *G. parasuis* strain 29755 genome (NCBI: NZ_CP021644.1) (8). The fastq files were respectively combined for each biological sample, as each biological replicate was run on two different lanes. The read counts per gene were calculated by using HTSeq-count 2.0.2 (9). The count files were imported into Galaxy, and DESeq2 was used to perform the differential expression analysis with the median of ratios normalization method and parametric fit type settings (10). One biological replicate from joint fluid was excluded from the analysis, as it was shown to be an outlier from all other samples in a PCA plot. The tabular output files were exported from Galaxy and imported into R v. 4.1.2 for further analysis (11). The data was filtered to require an adjusted p-value of 0.05 and a log_2_-fold change >1. Supplementary Tables S1 and S2 contain the resultant output. COG data was assigned using COGclassifier (https://github.com/moshi4/COGclassifier, accessed 5/05/2023). The word “gene” is used interchangeably with “gene id” when discussing the numerical differences for items differentially expressed.

## Results

### Differential expression in acellular host fluid

**Figure 1:**
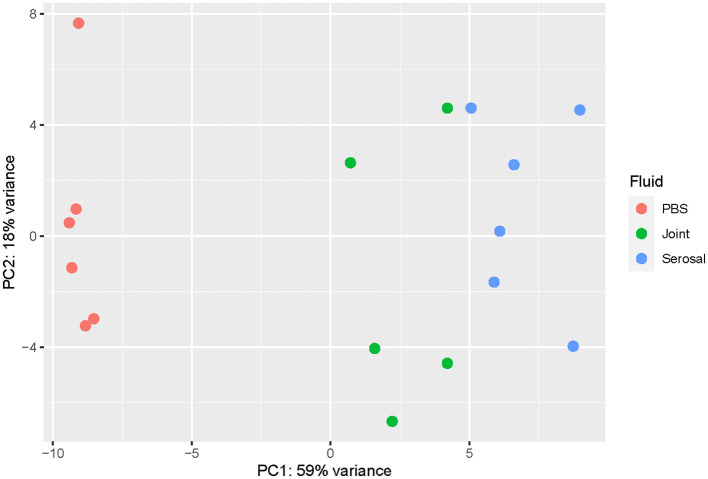
Principal component analysis (PCA) plot evaluating the variance among samples at the 3h timepoint. The transcriptome of *G. parasuis* strain 29755 when inoculated into phosphate buffered saline (PBS) (coral), joint fluid (green), or serosal fluid (blue). The joint and serosal fluids separated from PBS, the negative control.

**Figure 2:**
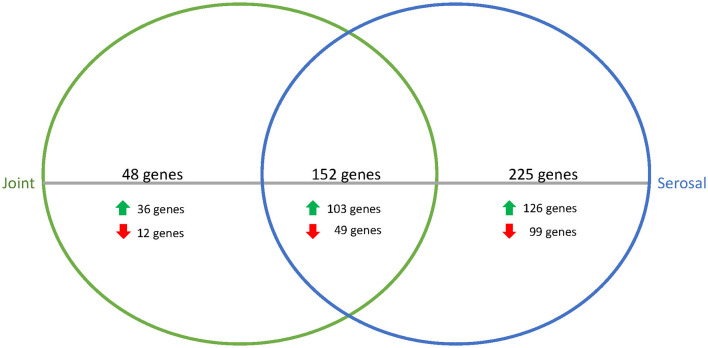
Venn diagram of differentially expressed genes in joint (green) or serosal (blue) fluid compared to PBS at the 3h timepoint. The serosal fluid had more differentially expressed genes than the joint fluid, but there was an overlap of 152 genes. The majority of the differentially expressed genes were upregulated in the host fluid compared to PBS.

When comparing the joint and serosal fluids to PBS at the 3h timepoint, the isolates grown in either of the host fluids clustered separately from PBS (Figure 1). There were 425 genes that were differentially expressed in any host fluid compared to PBS (Figure 2, Supplementary Table S1). However, more genes were differentially expressed in the serosal fluid than the joint fluid when compared to PBS (Figure 2, Supplementary Table S1, Supplementary Figure S1). Of the 425 differentially expressed genes in host fluid compared to PBS, only four were previously described virulence-associated genes. Gene *glgB*, a glycogen metabolism and synthesis gene implicated in biofilm formation (12), was upregulated in both the joint and serosal fluid compared to PBS. Gene *glmM*, a virulence-associated gene, which is used for cell wall biosynthesis and may contribute to bacterial resistance against macrolide antibiotics (13), was downregulated in the serosal fluid compared to PBS, but *glmM* was not differentially expressed in the joint fluid. Gene *nqrE*, a transport protein encoding gene (14), was upregulated in serosal fluid compared to PBS, but it was not differentially expressed in the joint fluid compared to PBS. Gene *uhpT*, a transport protein encoding gene (15), was downregulated in the serosal fluid compared to PBS, but the gene was not differentially expressed in the joint fluid compared to PBS.

### Clusters of orthologous genes (COGs)

The 425 differentially expressed genes at the 3h timepoint were assigned to different COG categories. The genes differentially expressed in serosal fluid compared to PBS and not in joint fluid compared to PBS were assigned most often to translation, ribosomal structure, and biogenesis (*n* = 19), amino acid transport and metabolism (*n* = 17), mobilome (*n* = 13), cell wall/membrane/envelope (*n* = 12), coenzyme transport and metabolism (*n* = 11), and replication, recombination and repair (*n* = 11). The most prevalent COG assignment for differentially expressed genes in the joint fluid compared to PBS and not in the serosal fluid compared to PBS were assigned to translation, ribosomal structure and biogenesis (*n* = 11).

To assess the similarities among the joint and serosal fluids compared to PBS, the inferred functions of the 152 genes were queried (Figure 2, Table 1). The majority of differentially expressed translation, ribosomal structure, and biogenesis (95%) and carbohydrate and metabolism (77%) genes were upregulated in the joint and serosal fluid compared to PBS (Table 1). Alternatively, the majority of the differentially expressed cell wall, membrane, and envelope biogenesis genes (73%) were downregulated in serosal and joint fluid compared to PBS (Table 1).

**Table 1:** COG assignments for differentially expressed genes shared between the joint and serosal fluid at 3h compared to PBS.

**COG**	**Upregulated**	**Downregulated**	**Total**
Translation, ribosomal structure and biogenesis	21	1	22
Carbohydrate transport and metabolism	10	3	13
Cell wall/membrane/envelope biogenesis	3	8	11
Function unknown	6	5	11
Mobilome: prophages, transposons	6	3	9
Energy production and conversion	6	2	8
Transcription	5	3	8
Replication, recombination and repair	5	3	8
Coenzyme transport and metabolism	6	1	7
Amino acid transport and metabolism	4	2	6
Defense mechanisms	4	2	6
General function prediction only	3	2	5
All other assignments	7	5	12
Unassigned	17	9	26
**Total**	**103**	**49**	**152**

Any COG with fewer than five total assignments is included in the category “All other assignments.” The COGs encompassed in this grouping include RNA processing and modification, chromatin structure and dynamics; cell cycle control, cell division, chromosome partitioning; nuclear structure; signal transduction mechanisms; cell motility; cytoskeleton; extracellular structures; and secondary metabolites biosynthesis, transport and catabolism. “Unassigned” refers to the number of genes that were not provided any COG assignment.

### Differential expression in acellular fluid during time progression

**Figure 3:**
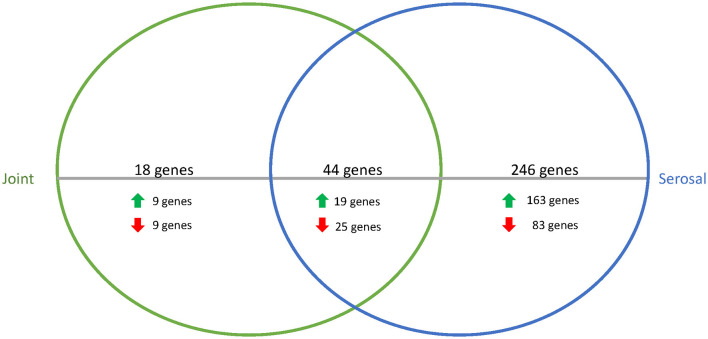
Venn diagram of differentially expressed genes in joint (green) or serosal (blue) fluid when the 12h timepoint was compared to the 3h timepoint. The serosal fluid had more differentially expressed genes than the joint fluid when the timepoints were compared, but there was an overlap of 44 genes that changed over time. The majority of the differentially expressed genes were upregulated at 12h compared to 3h (data in Supplementary Table S2).

To better understand how *G. parasuis* gene expression may differ as infection progresses, the differences in the transcriptome in acellular joint and serosal fluid were compared between the 3 and 12h timepoints. When comparing the differential expression of the joint and serosal fluids at 12h to 3h, there were 308 genes that were differentially expressed among the joint and serosal fluids (Supplementary Table S2, Supplementary Figure S2). Similar to the 3h serosal to PBS and 3h joint to PBS comparison, the 12 to 3h serosal comparison had more genes differentially expressed than the 12 to 3h joint fluid comparison, and most differentially expressed genes were upregulated (Figure 3). Unlike the differential expression at 3h (Table 1), there was a similar number of genes in the translation, ribosomal structure, and biogenesis COG that were upregulated and downregulated at 12h in both fluids compared to 3h (Table 2). Minimal differences in number were observed among the cell wall/membrane/envelope biogenesis (4 upregulated, 2 downregulated), mobilome: prophages, transposons (0 upregulated, 2 downregulated), Energy production and conversion (0 upregulated, 2 downregulated), Carbohydrate transport and metabolism (0 upregulated, 2 downregulated), and Transcription (0 upregulated, 2 downregulated) (Table 2).

**Table 2:** COG assignments of differentially expressed genes shared between the joint fluid and serosal fluid when comparing 12 to 3h.

**COG**	**Upregulated**	**Downregulated**	**Total**
Translation, ribosomal structure and biogenesis	4	4	8
Cell wall/membrane/envelope biogenesis	4	2	6
Defense mechanisms	1	2	3
Transcription	0	2	2
Replication, recombination and repair	1	1	2
Mobilome: prophages, transposons	0	2	2
Energy production and conversion	0	2	2
Carbohydrate transport and metabolism	0	2	2
Coenzyme transport and metabolism	1	1	2
Cell cycle control, cell division, chromosome partitioning	1	0	1
Amino acid transport and metabolism	1	0	1
Inorganic ion transport and metabolism	0	1	1
Unassigned	6	5	12
**Total**	**19**	**25**	**44**

### Nineteen differentially expressed genes shared in the two previous comparisons

Nineteen genes were observed to be differentially expressed both for the serosal and joint fluid compared to PBS at 3h and for both the joint and serosal fluid when comparing the 3-to-12h timepoints. Sixteen of the 19 genes were inversely differentially expressed (Table 3). The remaining three genes were either upregulated in all the comparisons (2 genes) or downregulated in all the comparisons (1 gene). These three genes were associated with the ribosome (Table 3). Fourteen of the 16 inversely differentially expressed genes were initially upregulated in the host fluids compared to the PBS at 3h, but these genes were downregulated at the 12h timepoint compared to the 3h expression levels in the joint and serosal fluid (Table 3). Interestingly, one of these gene IDs appeared exclusively in *G. parasuis* (B4U42_RS02905) when its amino acid (protein) and nucleic acid sequences were queried via BLAST (blastp, blastn, megablast, and PSI-BLAST) using the nr and nt databases, respectively (Table 3). Other notable CDS products were associated with iron acquisition or bacterial membrane (Table 3). These results suggest a small core of *G. parasuis* genes may be differentially expressed in stressful, host-like conditions.

**Table 3:** Genes with differential expression in both the 3h host fluids compared to PBS and the 12h host fluids compared to the 3h host fluids.

**Gene ID**	**Gene**	**12S_3S**	**12J_3J**	**3S_3N**	**3J_3N**	**Product**
B4U42_RS00415	NA	Down	Down	Up	Up	Heme ABC transporter ATP-binding protein
B4U42_RS00855	*hemH*	Down	Down	Up	Up	Ferrochelatase
B4U42_RS01770	*ung*	Down	Down	Up	Up	Uracil-DNA glycosylase
B4U42_RS02615	NA	Down	Down	Up	Up	Trm112 family protein
B4U42_RS02905	NA	Down	Down	Up	Up	Hypothetical protein
B4U42_RS03665	*kdsB*	Down	Down	Up	Up	3-deoxy-manno-octulosonate cytidylyltransferase
B4U42_RS04575	NA	Down	Down	Up	Up	RnfH family protein
B4U42_RS06875	*cspD*	Down	Down	Up	Up	Cold shock domain-containing protein CspD
B4U42_RS07235	*glgB*	Down	Down	Up	Up	1,4-alpha-glucan branching protein GlgB
B4U42_RS07705	*lacD*	Down	Down	Up	Up	Tagatose-bisphosphate aldolase
B4U42_RS09240	*tusA*	Down	Down	Up	Up	Sulfurtransferase TusA
B4U42_RS09250	NA	Down	Down	Up	Up	LysR family transcriptional regulator
B4U42_RS10880	NA	Down	Down	Up	Up	YfhL family 4Fe-4S dicluster ferredoxin
B4U42_RS11625	NA	Down	Down	Up	Up	ISAs1 family transposase
B4U42_RS04415	*mlaD*	Up	Up	Down	Down	Heme ABC transporter ATP-binding protein
B4U42_RS07725	*mepM*	Up	Up	Down	Down	Murein DD-endopeptidase MepM
B4U42_RS11965	*rpsQ*	Up	Up	Up	Up	30S ribosomal protein S17
B4U42_RS12010	*rplC*	Up	Up	Up	Up	50S ribosomal protein L3
B4U42_RS01955	*ssrS*	Down	Down	Down	Down	6S rRNA

The comparisons are the 12h serosal to 3h serosal comparison (12S_3S), the 12h joint to the 3h joint comparison (12J_3J), the 3h serosal compared to PBS (3S_3N), and the 3h joint compared to PBS (3J_3N). Of the 19 differentially expressed genes, sixteen CDS were inversely differentially expressed. Seven of the 16 did not have gene names associated with the sequence (NA). The non-inversely expressed genes were associated with the ribosome.

## Discussion

*G. parasuis* disease is characterized by polyserositis, meningitis, and polyarthritis (1). Although previous work has investigated transcriptional differences in *G. parasuis* under various host and host-like conditions (3–5, 16), there are still many questions surrounding the mechanism by which *G. parasuis* causes disease, specifically serositis and arthritis. In this study, we evaluated transcriptomic changes of *G. parasuis* when it was exposed to acellular serosal or joint fluid to better understand *G. parasuis* changes in the host environment. We evaluated an early timepoint (3h) compared to our standard challenge media (PBS), which controlled for temperature but not the potential for differences in replication or stress. We also evaluated at a later timepoint (12h) to better understand the progression of infection.

In this study, we found a core of 19 genes that were differentially expressed in the serosal and joint fluid compared to PBS at 3h and were differentially expressed when 12h was compared to 3h. Sixteen of these 19 genes were inversely expressed in the 12 to 3h comparison vs. the 3h host fluid to PBS comparison. Given the relatively small list of genes differentially expressed under the various conditions, it suggests that there may be a core set of differentially expressed genes that *G. parasuis* modulates when adapting to a host niche. Genes *mepM* and *mlaD* were downregulated in the host fluid compared to the PBS, but they were upregulated when comparing 12h to 3h. MepM is a peptidoglycan hydrolase, which is involved in peptidoglycan synthesis. Experimentally, in *Escherichia coli*, MepM is regulated in an amino acid availability dependent manner, as the presence of glutamate in a medium increased the level of MepM (17). MlaD, is part of the OmpC-Mla (maintenance of lipid asymmetry) system, which is responsible for moving phospholipids from the outer to the inner membrane for Gram negatives, like *G. parasuis* (18). Like *mepM* and *mlaD*, a majority of the differentially expressed cell wall, membrane, and envelope biogenesis genes were downregulated in the host fluids compared to the PBS at 3h. The downregulation of these membrane genes may have occurred as the bacteria adapted to a harsh, nutrient limited environment (PBS).

Genes *cspD, glgB, hemH, kdsB, lacD, tusA, ung*, and seven unnamed genes were upregulated in the acellular host fluid compared to PBS, but they were downregulated when comparing 12h to 3h. Regarding *cspD*, Bello-Ortí et al. (5) found the gene encoding cold shock-like protein CspD in a virulent *G. parasuis* strain to be downregulated in a lung *ex vivo* system compared to an agar plate. The differing results may be a result of the comparisons made between these studies. For example, this current study compared, at 3h, to PBS, a nutrient limited environment, instead of an agar plate. However, these combined results suggest transcription changes of *cspD* may be important for *G. parasuis* infection. In *G. parasuis*, gene *kdsB* is essential for the synthesis of lipid A (19), and ferrochelatase, encoded by *hemH*, is the terminal enzyme of the heme biosynthetic pathway. These genes may be important during the early stages of infection when replication is increasing to ensure cell membrane integrity and iron availability. Additionally, an unnamed LysR-type transcriptional regulator (B4U42_RS09250) was upregulated in the host fluids compared to the PBS, but it was downregulated when comparing 12h to 3h. LysR-type transcriptional regulators are involved in essential cellular processes, as they are DNA-binding proteins involved in the transcriptional regulation of tasks including amino acid biosynthesis, cell division, central metabolism, oxidative stress, quorum sensing, motility, and virulence (20). The function of the unnamed LysR-type transcriptional regulator remains unknown for *G. parasuis*. However, more differentially expressed carbohydrate transport and metabolism genes were upregulated in the host fluid compared to the PBS at 3h. Similarly, most differentially expressed translation, ribosomal structure, and biogenesis genes were upregulated in the host fluid compared to PBS at 3h. The appearance of the seven unnamed genes in both the 3h fluids and 12 to 3h datasets suggests that further characterization of *G. parasuis* genes is needed (Table 3). Specifically, it would be interesting to better understand if these unnamed genes encode products that assist *G. parasuis* during the infection process. The role of B4U42_RS02905 is of particular interest, as it appears to be specific to *G. parasuis*.

For both the 3h host to PBS and 12-to-3h comparisons, the serosal fluid had more differentially expressed, and upregulated, genes than the joint fluid. Although we cannot confirm any particular reason why this occurred, there are many possible factors, including the potential complexity of the serosal cavity compared to the joint cavity or the composition of joint fluid as compared to serosal fluid. In this study, we also examined the differential expression of the virulence-associated genes indicated by Wan et al. (21) as pathogenic factors. We found that only *glgB, glmM, nqrE*, and *uhpT* were differentially regulated in the joint or serosal fluid at 3h compared to PBS. The gene *glgB* was upregulated in both host fluids and downregulated at 12h compared to 3h, which suggests that the gene may be important early in replication. The other three genes (*glmM, nqrE*, and *uhpT*) were differentially expressed only in serosal fluid compared to PBS at 3h. These results are consistent with the overall results of this study, which found more genes differentially expressed in the serosal fluid than the joint fluid when compared to PBS.

The identification of four differentially expressed virulence associated genes (*glgB, glmM, nqrE*, and *uhpT)* differs from Bello-Ortí et al. (5) who found 11 different virulence factors and key pathways to be upregulated when the strain Nagasaki was incubated in lung explants and in the lung 2 h after intratracheal inoculation. Bello-Ortí et al. (5) found an ABC transporter, permease protein upregulated in the lung systems. We found this gene was differentially expressed, as it was downregulated at 12h compared to 3h in the serosal fluid (HPNK_10441/B4U42_00670). However, there was no significant differential expression at 3h between the host fluids compared to PBS, which suggests this significance may be comparison specific. Bello-Ortí et al. (5) also found a key pathway gene for 6-pyruvoyl tetrahydrobiopterin synthase to be upregulated. We also observed differential expression for 6-pyruvoyl tetrahydrobiopterin synthase, as it was upregulated in the serosal fluid compared to PBS at the 3h timepoint (HPNK_03728/B4U42_10060). The limited number of virulence-associated genes differentially expressed may have been due to experimental design, as temperature is well known to regulate virulence genes in bacteria. Alternatively, the limited number of virulence-associated genes observed may have been reduced, as the joint and serosal fluid were both acellular due to filtration prior to inoculation with bacteria, and some of the virulence genes upregulated in the Bello-Ortí study (5) may be associated with cell-cell interactions. Likewise, differences among the work by Bello-Ortí and this current work may be explained by the location inoculated (lung compared to the joint or serosal fluid, respectively), the difference in incubation time (2 h compared to 3 and 12, respectively), or strain used (Nagasaki compared to 29755, respectively). Nevertheless, this study demonstrates important interactions among *G. parasuis* strain 29755 and different host fluids. Further research is warranted to determine how *G. parasuis* responds during infection in the host.

Prior work by Metcalf and MacInnes (16) found that *G. parasuis* strain HP1185, a member of serogroup 5 like strain 29755, upregulated genes in response to iron restriction (16). For HP1185, *fba*, which encodes the metabolic enzyme fructose-1,6-bisphosphate aldolase, was upregulated (16). Like HP1185, *fba* (RS09560) was differentially expressed for strain 29755, as it was upregulated in the joint and serosal fluid compared to the PBS at the 3h timepoint. Together, this suggests that *fba* appears to be differentially expressed under host-like conditions by serovar 5 strains.

Previous work by Hau et al. (3) found differences among the transcriptional profile of 29755 when grown on BHI-NAD-HS agar and in BHI-NAD-HS broth. Specifically, agar grown *G. parasuis* showed elevated expression of *vapD* and *ompA*, and broth grown *G. parasuis* showed elevated expression of proteases, including *rseP*. In this study, we did not grow 29755 in these media, but we observed that *rseP* was upregulated at 12h compared to 3h in serosal fluid (Supplementary Table S2), but no differential expression occurred among the different fluid types at 3h or from 12 to 3h in the joint fluid. We also observed that *ompA* was downregulated for the serosal fluid compared to the negative control (PBS) at 3h (Supplementary Table S1). No differential expression of *vapD* was observed (Supplementary Tables S1, S2). The results suggest that the differences in expression between agar and broth expression for strain 29755 cannot be easily translated to joint or serosal fluid from a host.

Here, we presented data indicating the differences in transcription between *G. parasuis* grown for 3h in acellular joint fluid and acellular serosal fluid compared to PBS and the differences between the joint or serosal fluids after incubating for another nine h (12h total). This study found 16 inversely expressed genes, of which 7 were unnamed, in all compared datasets, which merit further attention. Likewise, the function of the eleven differentially expressed genes with an unknown function shared among the acellular joint and serosal fluid compared to PBS at the 3h mark should be investigated. An investigation into these hypothetical genes may further knowledge about the pathogenesis of *G. parasuis*, which may be useful in developing interventions against Glässer's disease.

## Data Availability

The datasets presented in this study can be found in online repositories. The names of the repository/repositories and accession number(s) can be found below: https://www.ncbi.nlm.nih.gov/, PRJNA1030322.

## Supplementary material

Supplementary Figure S1 Heatmap for all differentially expressed genes for the joint and serosal fluids compared to PBS at 3h **(A)** and heatmap for unnamed differentially expressed genes for the joint and serosal fluids compared to PBS at 3h **(B)**. Data is in Supplementary Table S1.

Supplementary Figure S2 Heatmap for all differentially expressed genes for the joint and serosal fluids when comparing 12h to 3h **(A)** and heatmap for unnamed differentially expressed genes for the joint and serosal fluids when comparing 12h to 3h **(B)**. Data is in Supplementary Table S2.
