# Supplementary figures and images for: Transcriptional profile of Glaesserella parasuis in swine serosal and joint fluids
Source: Front Vet Sci. 2025 Apr 25;12:1452973. doi: 10.3389/fvets.2025.1452973 (PMC12063495; doi:10.3389/fvets.2025.1452973)

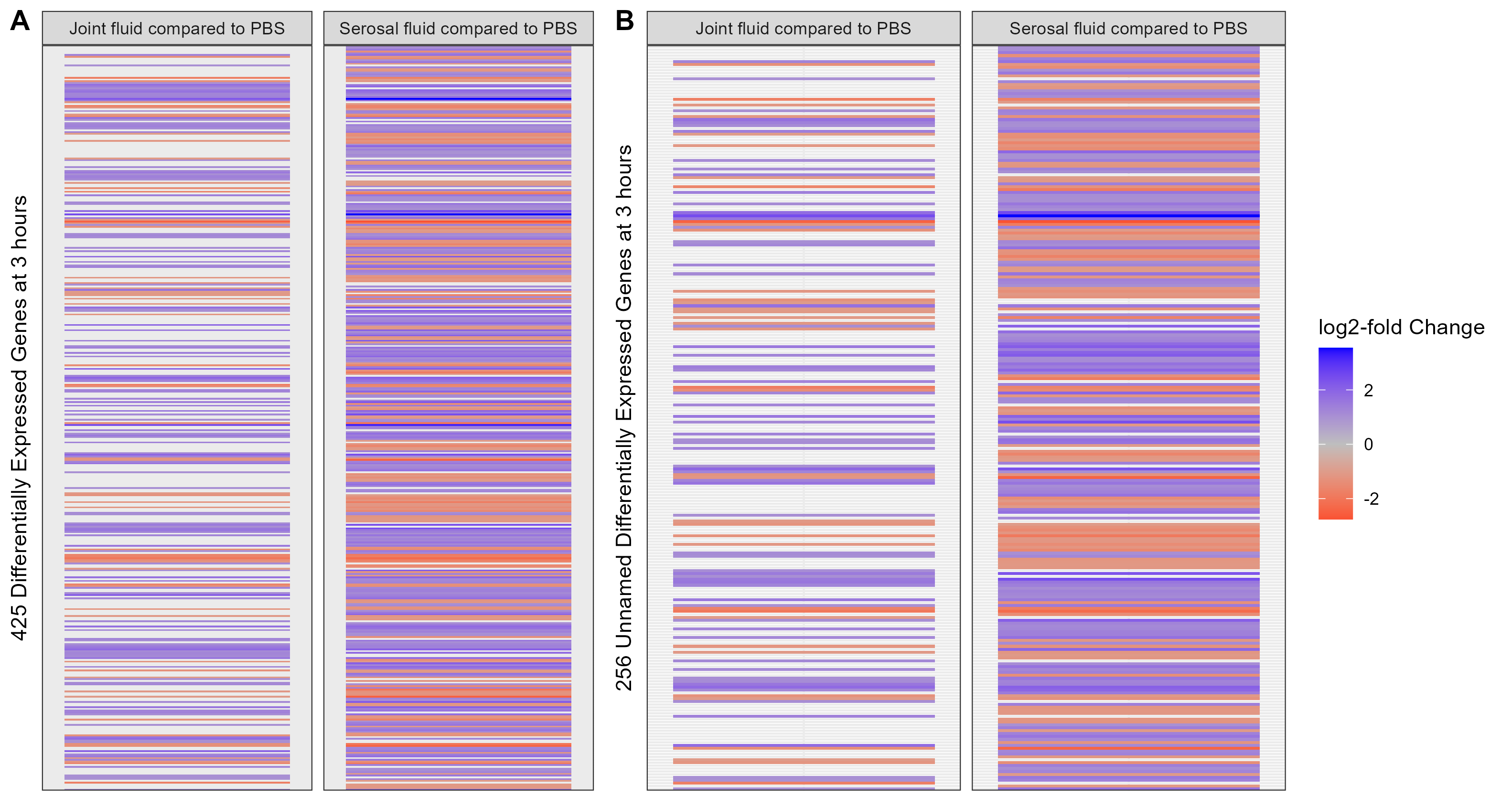

Supplement: Supplementary Figure S1 — Heatmap for all differentially expressed genes for the joint and serosal fluids compared to PBS at 3h (A) and heatmap for unnamed differentially expressed genes for the joint and serosal fluids compared to PBS at 3h (B). Data is in Supplementary Table S1. [file Image_1.png]

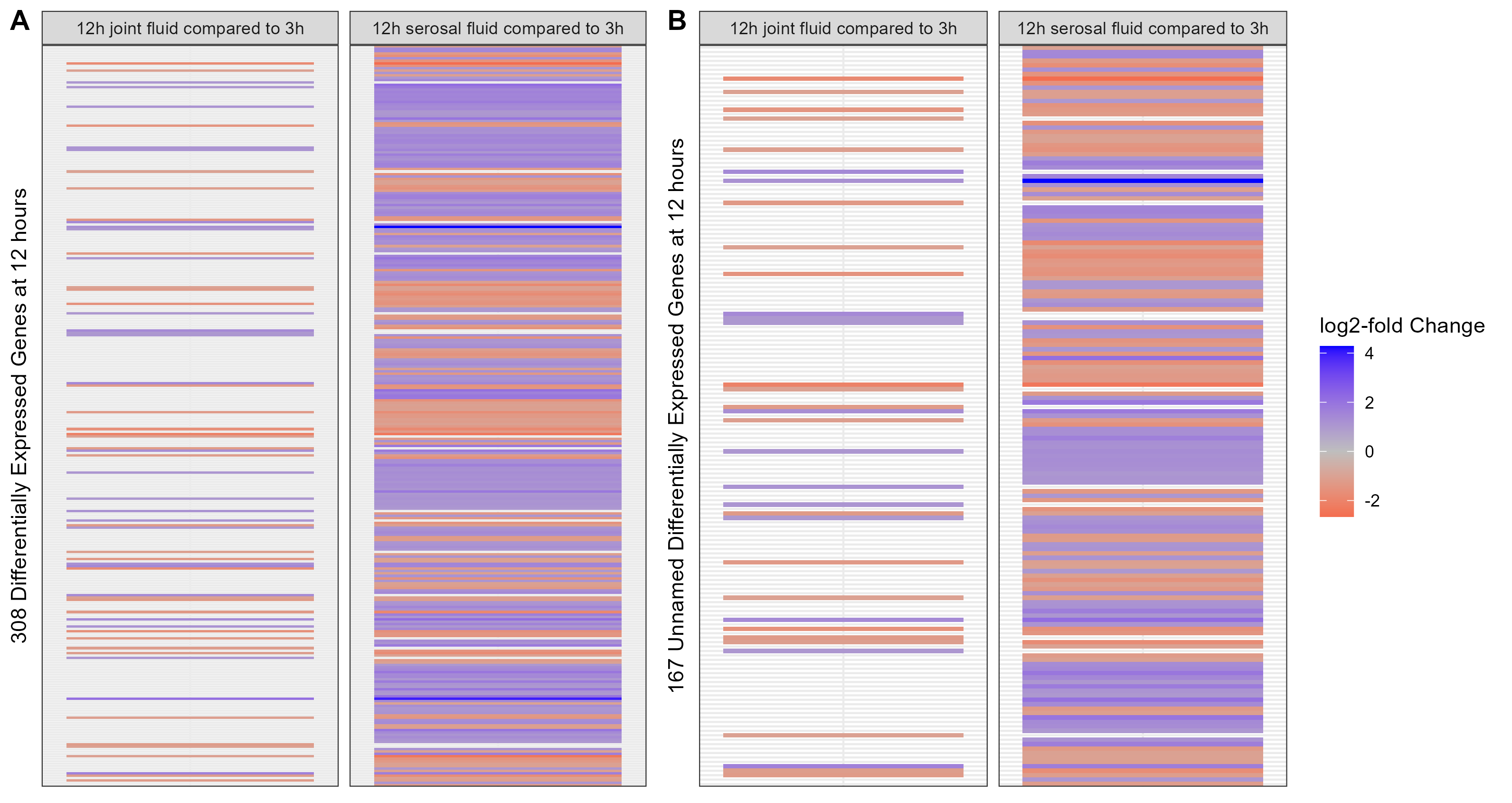

Supplement: Supplementary Figure S2 — Heatmap for all differentially expressed genes for the joint and serosal fluids when comparing 12h to 3h (A) and heatmap for unnamed differentially expressed genes for the joint and serosal fluids when comparing 12h to 3h (B). Data is in Supplementary Table S2. [file Image_2.png]
